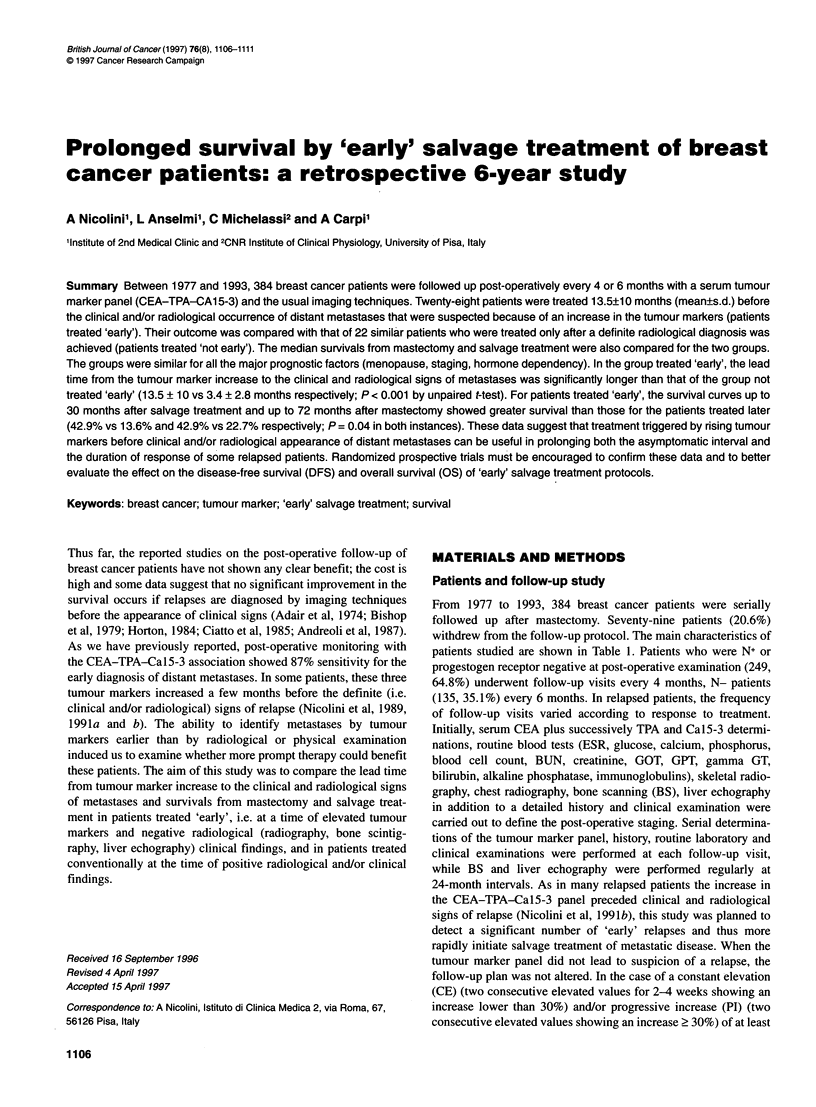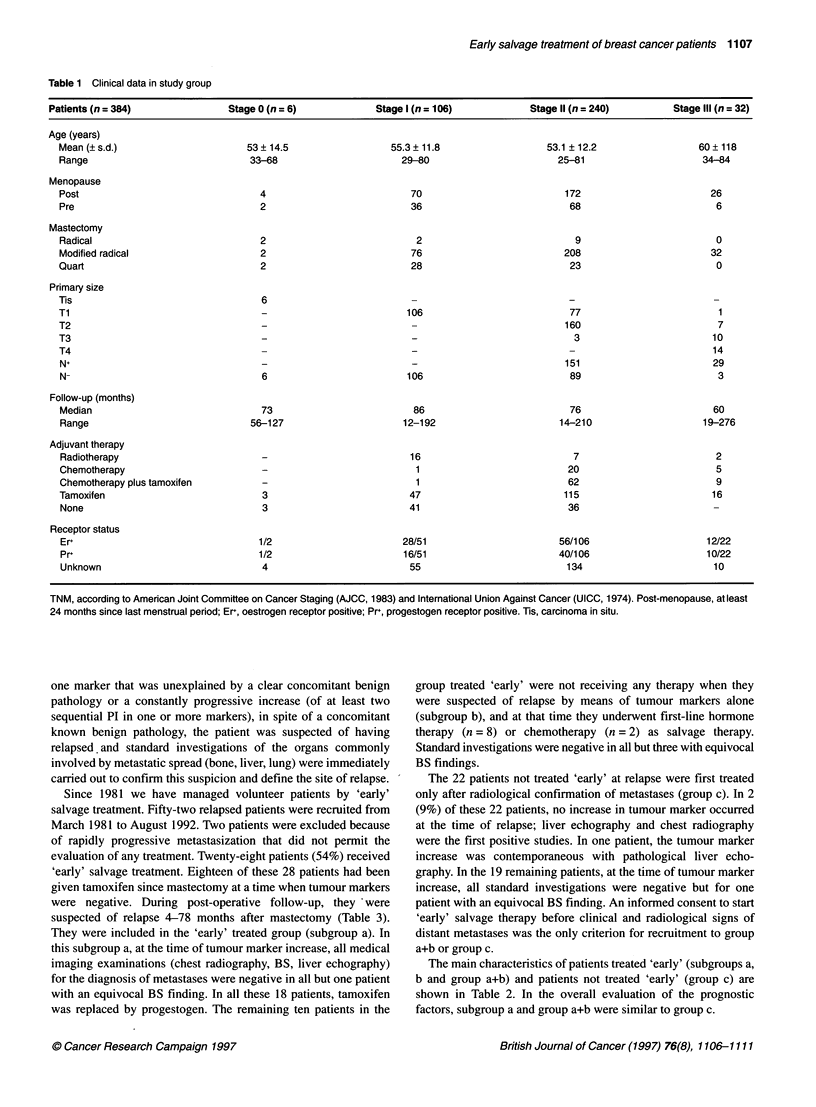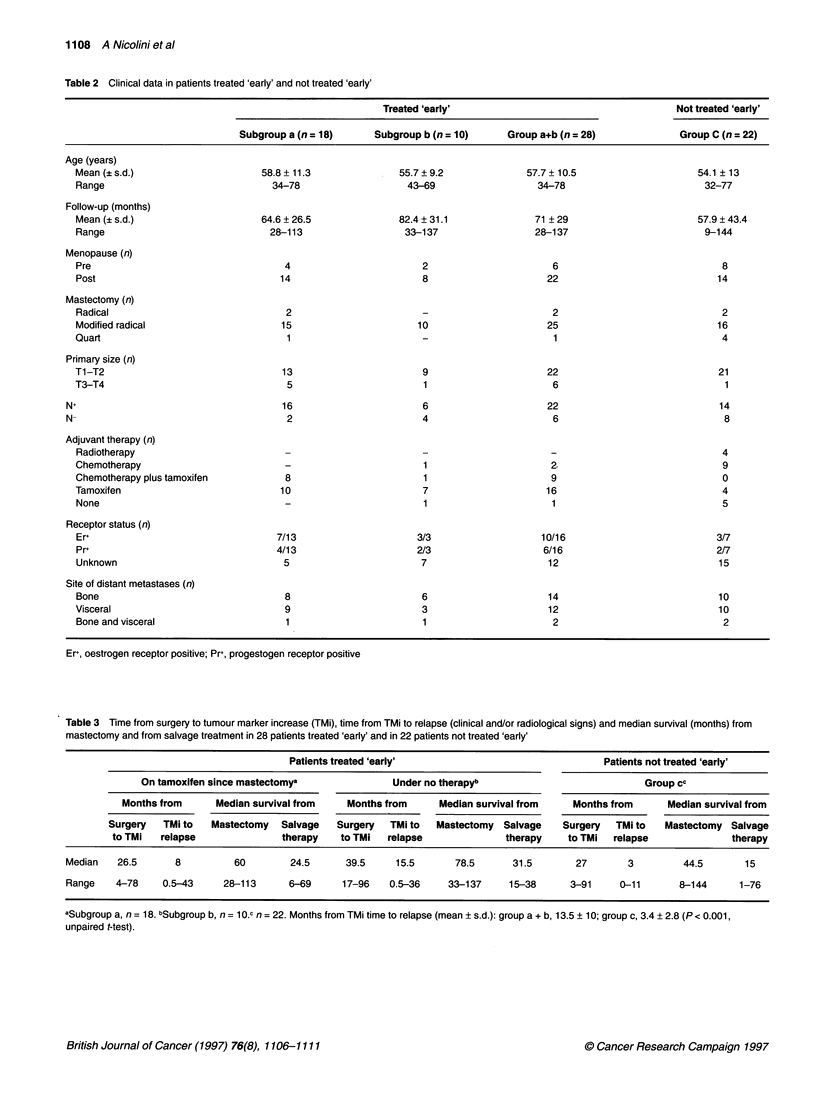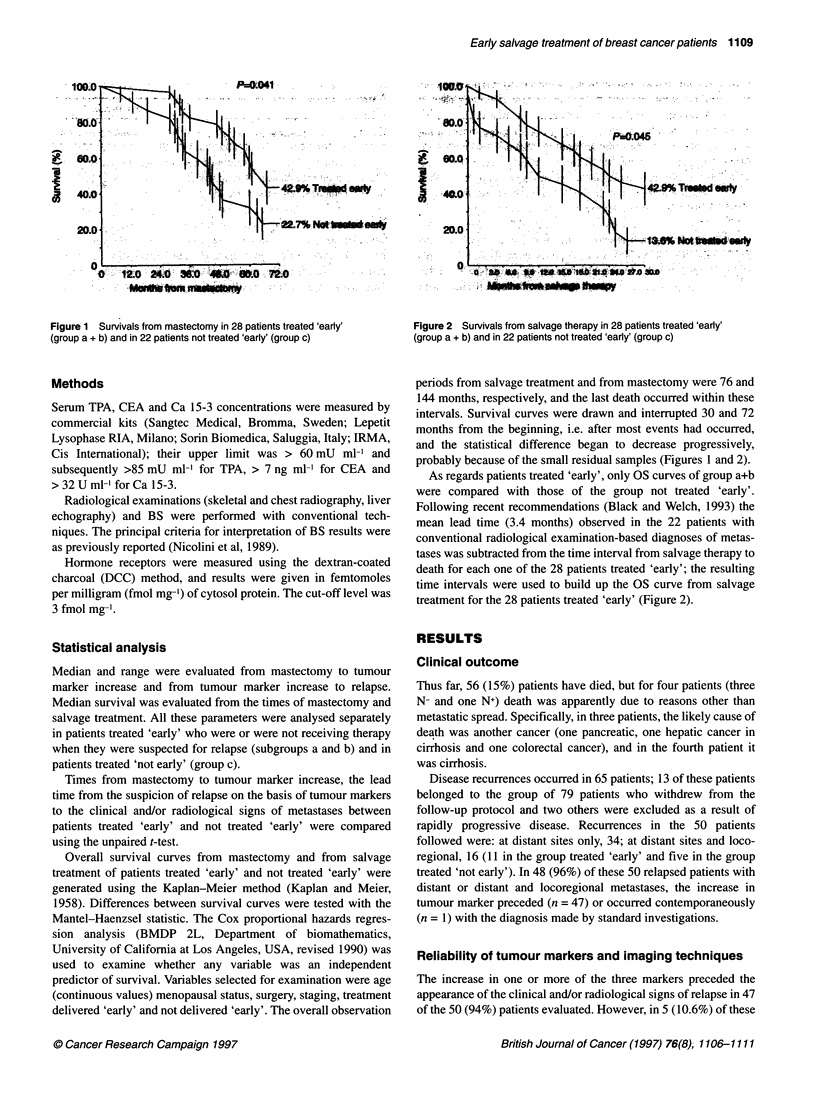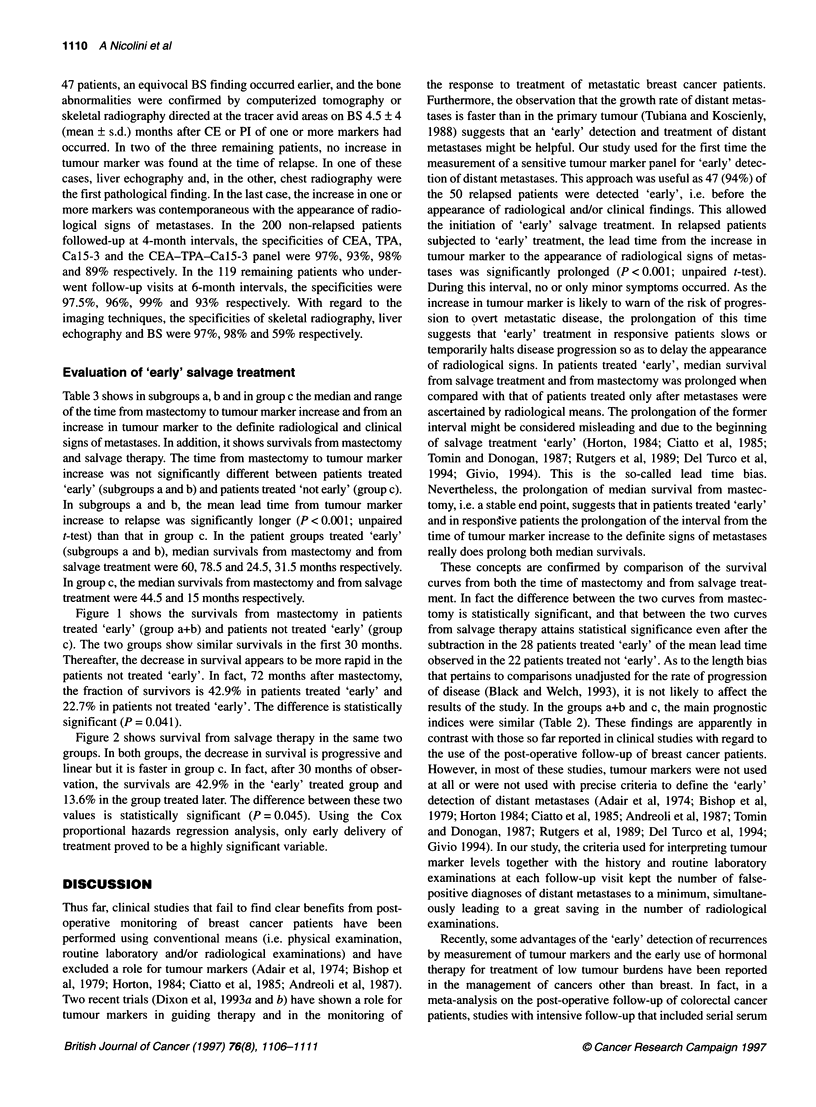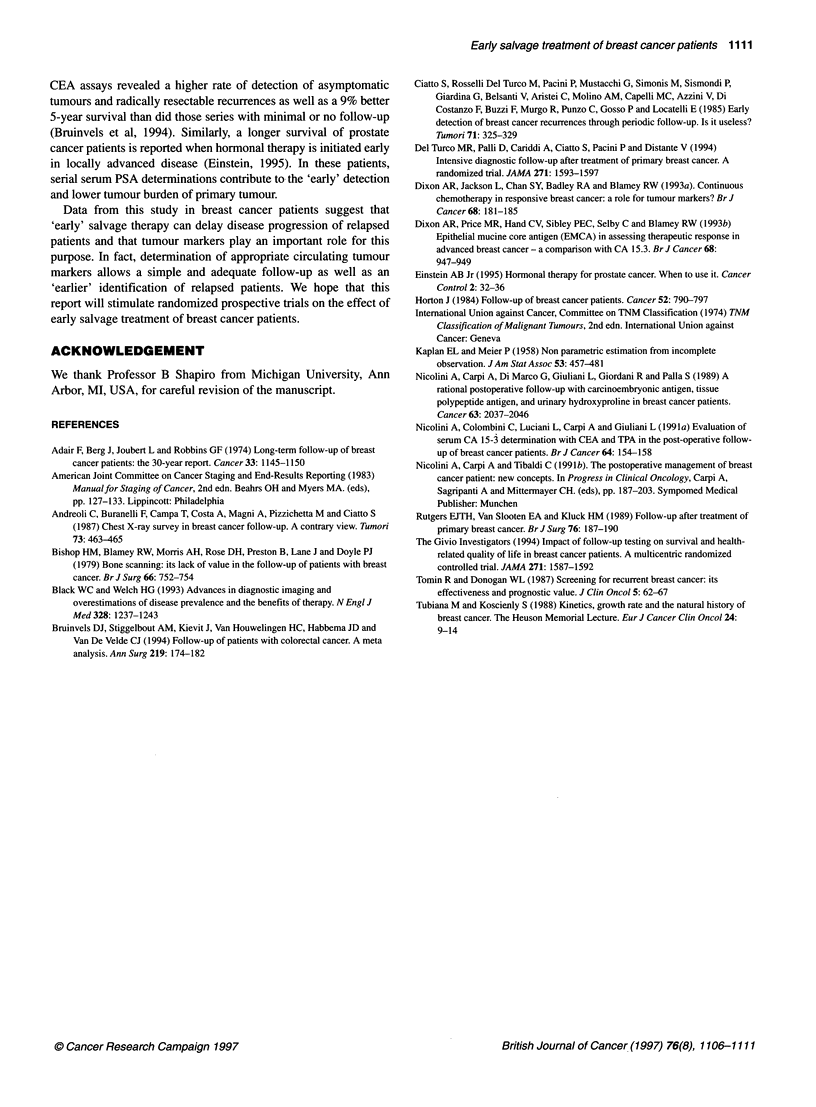# Prolonged survival by 'early' salvage treatment of breast cancer patients: a retrospective 6-year study.

**DOI:** 10.1038/bjc.1997.515

**Published:** 1997

**Authors:** A. Nicolini, L. Anselmi, C. Michelassi, A. Carpi

**Affiliations:** Institute of 2nd Medical Clinic, University of Pisa, Italy.

## Abstract

Between 1977 and 1993, 384 breast cancer patients were followed up post-operatively every 4 or 6 months with a serum tumour marker panel (CEA-TPA-CA15-3) and the usual imaging techniques. Twenty-eight patients were treated 13.5 +/- 10 months (mean +/- s.d.) before the clinical and/or radiological occurrence of distant metastases that were suspected because of an increase in the tumour markers (patients treated 'early'). Their outcome was compared with that of 22 similar patients who were treated only after a definite radiological diagnosis was achieved (patients treated 'not early'). The median survivals from mastectomy and salvage treatment were also compared for the two groups. The groups were similar for all the major prognostic factors (menopause, staging, hormone dependency). In the group treated 'early', the lead time from the tumour marker increase to the clinical and radiological signs of metastases was significantly longer than that of the group not treated 'early' (13.5 +/- 10 vs 3.4 +/- 2.8 months respectively; P < 0.001 by unpaired t-test). For patients treated 'early', the survival curves up to 30 months after salvage treatment and up to 72 months after mastectomy showed greater survival than those for the patients treated later (42.9% vs 13.6% and 42.9% vs 22.7% respectively; P = 0.04 in both instances). These data suggest that treatment triggered by rising tumour markers before clinical and/or radiological appearance of distant metastases can be useful in prolonging both the asymptomatic interval and the duration of response of some relapsed patients. Randomized prospective trials must be encouraged to confirm these data and to better evaluate the effect on the disease-free survival (DFS) and overall survival (OS) of 'early' salvage treatment protocols.


British Journal of Cancer (1997) 76(8), 1106–1111
© 1997 Cancer Research Campaign

### Prolonged survival by 'early' salvage treatment of breast cancer patients: a retrospective 6-year study

A Nicolini[1], L Anselmi[1], C Michelassi[2] and A Carpi[1]

[1]Institute of 2nd Medical Clinic and [2]CNR Institute of Clinical Physiology, University of Pisa, Italy

**Summary** Between 1977 and 1993, 384 breast cancer patients were followed up post-operatively every 4 or 6 months with a serum tumour marker panel (CEA–TPA–CA15-3) and the usual imaging techniques. Twenty-eight patients were treated 13.5±10 months (mean±s.d.) before the clinical and/or radiological occurrence of distant metastases that were suspected because of an increase in the tumour markers (patients treated 'early'). Their outcome was compared with that of 22 similar patients who were treated only after a definite radiological diagnosis was achieved (patients treated 'not early'). The median survivals from mastectomy and salvage treatment were also compared for the two groups. The groups were similar for all the major prognostic factors (menopause, staging, hormone dependency). In the group treated 'early', the lead time from the tumour marker increase to the clinical and radiological signs of metastases was significantly longer than that of the group not treated 'early' (13.5 ± 10 vs 3.4 ± 2.8 months respectively; $P < 0.001$ by unpaired $t$-test). For patients treated 'early', the survival curves up to 30 months after salvage treatment and up to 72 months after mastectomy showed greater survival than those for the patients treated later (42.9% vs 13.6% and 42.9% vs 22.7% respectively; $P = 0.04$ in both instances). These data suggest that treatment triggered by rising tumour markers before clinical and/or radiological appearance of distant metastases can be useful in prolonging both the asymptomatic interval and the duration of response of some relapsed patients. Randomized prospective trials must be encouraged to confirm these data and to better evaluate the effect on the disease-free survival (DFS) and overall survival (OS) of 'early' salvage treatment protocols.

**Keywords**: breast cancer; tumour marker; 'early' salvage treatment; survival

Thus far, the reported studies on the post-operative follow-up of breast cancer patients have not shown any clear benefit; the cost is high and some data suggest that no significant improvement in the survival occurs if relapses are diagnosed by imaging techniques before the appearance of clinical signs (Adair et al, 1974; Bishop et al, 1979; Horton, 1984; Ciatto et al, 1985; Andreoli et al, 1987). As we have previously reported, post-operative monitoring with the CEA–TPA–Ca15-3 association showed 87% sensitivity for the early diagnosis of distant metastases. In some patients, these three tumour markers increased a few months before the definite (i.e. clinical and/or radiological) signs of relapse (Nicolini et al, 1989, 1991*a* and *b*). The ability to identify metastases by tumour markers earlier than by radiological or physical examination induced us to examine whether more prompt therapy could benefit these patients. The aim of this study was to compare the lead time from tumour marker increase to the clinical and radiological signs of metastases and survivals from mastectomy and salvage treatment in patients treated 'early', i.e. at a time of elevated tumour markers and negative radiological (radiography, bone scintigraphy, liver echography) clinical findings, and in patients treated conventionally at the time of positive radiological and/or clinical findings.

*Received 16 September 1996*
*Revised 4 April 1997*
*Accepted 15 April 1997*

*Correspondence to:* A Nicolini, Istituto di Clinica Medica 2, via Roma, 67, 56126 Pisa, Italy

### MATERIALS AND METHODS

### Patients and follow-up study

From 1977 to 1993, 384 breast cancer patients were serially followed up after mastectomy. Seventy-nine patients (20.6%) withdrew from the follow-up protocol. The main characteristics of patients studied are shown in Table 1. Patients who were $N^+$ or progestogen receptor negative at post-operative examination (249, 64.8%) underwent follow-up visits every 4 months, N– patients (135, 35.1%) every 6 months. In relapsed patients, the frequency of follow-up visits varied according to response to treatment. Initially, serum CEA plus successively TPA and Ca15-3 determinations, routine blood tests (ESR, glucose, calcium, phosphorus, blood cell count, BUN, creatinine, GOT, GPT, gamma GT, bilirubin, alkaline phosphatase, immunoglobulins), skeletal radiography, chest radiography, bone scanning (BS), liver echography in addition to a detailed history and clinical examination were carried out to define the post-operative staging. Serial determinations of the tumour marker panel, history, routine laboratory and clinical examinations were performed at each follow-up visit, while BS and liver echography were performed regularly at 24-month intervals. As in many relapsed patients the increase in the CEA–TPA–Ca15-3 panel preceded clinical and radiological signs of relapse (Nicolini et al, 1991*b*), this study was planned to detect a significant number of 'early' relapses and thus more rapidly initiate salvage treatment of metastatic disease. When the tumour marker panel did not lead to suspicion of a relapse, the follow-up plan was not altered. In the case of a constant elevation (CE) (two consecutive elevated values for 2–4 weeks showing an increase lower than 30%) and/or progressive increase (PI) (two consecutive elevated values showing an increase ≥ 30%) of at least

**Table 1** Clinical data in study group

| Patients ($n$ = 384) | Stage 0 ($n$ = 6) | Stage I ($n$ = 106) | Stage II ($n$ = 240) | Stage III ($n$ = 32) |
|---|---|---|---|---|
| Age (years) | | | | |
| Mean (± s.d.) | 53 ± 14.5 | 55.3 ± 11.8 | 53.1 ± 12.2 | 60 ± 118 |
| Range | 33–68 | 29–80 | 25–81 | 34–84 |
| Menopause | | | | |
| Post | 4 | 70 | 172 | 26 |
| Pre | 2 | 36 | 68 | 6 |
| Mastectomy | | | | |
| Radical | 2 | 2 | 9 | 0 |
| Modified radical | 2 | 76 | 208 | 32 |
| Quart | 2 | 28 | 23 | 0 |
| Primary size | | | | |
| Tis | 6 | – | – | – |
| T1 | – | 106 | 77 | 1 |
| T2 | – | – | 160 | 7 |
| T3 | – | – | 3 | 10 |
| T4 | – | – | – | 14 |
| $N^+$ | – | – | 151 | 29 |
| $N^-$ | 6 | 106 | 89 | 3 |
| Follow-up (months) | | | | |
| Median | 73 | 86 | 76 | 60 |
| Range | 56–127 | 12–192 | 14–210 | 19–276 |
| Adjuvant therapy | | | | |
| Radiotherapy | – | 16 | 7 | 2 |
| Chemotherapy | – | 1 | 20 | 5 |
| Chemotherapy plus tamoxifen | – | 1 | 62 | 9 |
| Tamoxifen | 3 | 47 | 115 | 16 |
| None | 3 | 41 | 36 | – |
| Receptor status | | | | |
| $Er^+$ | 1/2 | 28/51 | 56/106 | 12/22 |
| $Pr^+$ | 1/2 | 16/51 | 40/106 | 10/22 |
| Unknown | 4 | 55 | 134 | 10 |

TNM, according to American Joint Committee on Cancer Staging (AJCC, 1983) and International Union Against Cancer (UICC, 1974). Post-menopause, at least 24 months since last menstrual period; $Er^+$, oestrogen receptor positive; $Pr^+$, progestogen receptor positive. Tis, carcinoma in situ.

one marker that was unexplained by a clear concomitant benign pathology or a constantly progressive increase (of at least two sequential PI in one or more markers), in spite of a concomitant known benign pathology, the patient was suspected of having relapsed and standard investigations of the organs commonly involved by metastatic spread (bone, liver, lung) were immediately carried out to confirm this suspicion and define the site of relapse.

Since 1981 we have managed volunteer patients by 'early' salvage treatment. Fifty-two relapsed patients were recruited from March 1981 to August 1992. Two patients were excluded because of rapidly progressive metastasization that did not permit the evaluation of any treatment. Twenty-eight patients (54%) received 'early' salvage treatment. Eighteen of these 28 patients had been given tamoxifen since mastectomy at a time when tumour markers were negative. During post-operative follow-up, they were suspected of relapse 4–78 months after mastectomy (Table 3). They were included in the 'early' treated group (subgroup a). In this subgroup a, at the time of tumour marker increase, all medical imaging examinations (chest radiography, BS, liver echography) for the diagnosis of metastases were negative in all but one patient with an equivocal BS finding. In all these 18 patients, tamoxifen was replaced by progestogen. The remaining ten patients in the group treated 'early' were not receiving any therapy when they were suspected of relapse by means of tumour markers alone (subgroup b), and at that time they underwent first-line hormone therapy ($n = 8$) or chemotherapy ($n = 2$) as salvage therapy. Standard investigations were negative in all but three with equivocal BS findings.

The 22 patients not treated 'early' at relapse were first treated only after radiological confirmation of metastases (group c). In 2 (9%) of these 22 patients, no increase in tumour marker occurred at the time of relapse; liver echography and chest radiography were the first positive studies. In one patient, the tumour marker increase was contemporaneous with pathological liver echography. In the 19 remaining patients, at the time of tumour marker increase, all standard investigations were negative but for one patient with an equivocal BS finding. An informed consent to start 'early' salvage therapy before clinical and radiological signs of distant metastases was the only criterion for recruitment to group a+b or group c.

The main characteristics of patients treated 'early' (subgroups a, b and group a+b) and patients not treated 'early' (group c) are shown in Table 2. In the overall evaluation of the prognostic factors, subgroup a and group a+b were similar to group c.

© Cancer Research Campaign 1997 

**Table 2** Clinical data in patients treated 'early' and not treated 'early'

| | Treated 'early' | | | Not treated 'early' |
|---|---|---|---|---|
| | Subgroup a ($n$ = 18) | Subgroup b ($n$ = 10) | Group a+b ($n$ = 28) | Group C ($n$ = 22) |
| Age (years) | | | | |
| Mean (± s.d.) | 58.8 ± 11.3 | 55.7 ± 9.2 | 57.7 ± 10.5 | 54.1 ± 13 |
| Range | 34–78 | 43–69 | 34–78 | 32–77 |
| Follow-up (months) | | | | |
| Mean (± s.d.) | 64.6 ± 26.5 | 82.4 ± 31.1 | 71 ± 29 | 57.9 ± 43.4 |
| Range | 28–113 | 33–137 | 28–137 | 9–144 |
| Menopause ($n$) | | | | |
| Pre | 4 | 2 | 6 | 8 |
| Post | 14 | 8 | 22 | 14 |
| Mastectomy ($n$) | | | | |
| Radical | 2 | – | 2 | 2 |
| Modified radical | 15 | 10 | 25 | 16 |
| Quart | 1 | – | 1 | 4 |
| Primary size ($n$) | | | | |
| T1–T2 | 13 | 9 | 22 | 21 |
| T3–T4 | 5 | 1 | 6 | 1 |
| $N^+$ | 16 | 6 | 22 | 14 |
| $N^-$ | 2 | 4 | 6 | 8 |
| Adjuvant therapy ($n$) | | | | |
| Radiotherapy | – | – | – | 4 |
| Chemotherapy | – | 1 | 2 | 9 |
| Chemotherapy plus tamoxifen | 8 | 1 | 9 | 0 |
| Tamoxifen | 10 | 7 | 16 | 4 |
| None | – | 1 | 1 | 5 |
| Receptor status ($n$) | | | | |
| $Er^+$ | 7/13 | 3/3 | 10/16 | 3/7 |
| $Pr^+$ | 4/13 | 2/3 | 6/16 | 2/7 |
| Unknown | 5 | 7 | 12 | 15 |
| Site of distant metastases ($n$) | | | | |
| Bone | 8 | 6 | 14 | 10 |
| Visceral | 9 | 3 | 12 | 10 |
| Bone and visceral | 1 | 1 | 2 | 2 |

$Er^+$, oestrogen receptor positive; $Pr^+$, progestogen receptor positive

**Table 3** Time from surgery to tumour marker increase (TMi), time from TMi to relapse (clinical and/or radiological signs) and median survival (months) from mastectomy and from salvage treatment in 28 patients treated 'early' and in 22 patients not treated 'early'

| | Patients treated 'early' | | | | | | | | Patients not treated 'early' | | | |
|---|---|---|---|---|---|---|---|---|---|---|---|---|
| | On tamoxifen since mastectomy[a] | | | | Under no therapy[b] | | | | Group c[c] | | | |
| | Months from | | Median survival from | | Months from | | Median survival from | | Months from | | Median survival from | |
| | Surgery to TMi | TMi to relapse | Mastectomy | Salvage therapy | Surgery to TMi | TMi to relapse | Mastectomy | Salvage therapy | Surgery to TMi | TMi to relapse | Mastectomy | Salvage therapy |
| Median | 26.5 | 8 | 60 | 24.5 | 39.5 | 15.5 | 78.5 | 31.5 | 27 | 3 | 44.5 | 15 |
| Range | 4–78 | 0.5–43 | 28–113 | 6–69 | 17–96 | 0.5–36 | 33–137 | 15–38 | 3–91 | 0–11 | 8–144 | 1–76 |

[a]Subgroup a, $n$ = 18. [b]Subgroup b, $n$ = 10.[c] $n$ = 22. Months from TMi time to relapse (mean ± s.d.): group a + b, 13.5 ± 10; group c, 3.4 ± 2.8 ($P$ < 0.001, unpaired $t$-test).

 *© Cancer Research Campaign 1997*

**Figure 1** Survivals from mastectomy in 28 patients treated 'early' (group a + b) and in 22 patients not treated 'early' (group c)

**Figure 2** Survivals from salvage therapy in 28 patients treated 'early' (group a + b) and in 22 patients not treated 'early' (group c)

### Methods

Serum TPA, CEA and Ca 15-3 concentrations were measured by commercial kits (Sangtec Medical, Bromma, Sweden; Lepetit Lysophase RIA, Milano; Sorin Biomedica, Saluggia, Italy; IRMA, Cis International); their upper limit was > 60 mU $ml^{-1}$ and subsequently >85 mU $ml^{-1}$ for TPA, > 7 ng $ml^{-1}$ for CEA and > 32 U $ml^{-1}$ for Ca 15-3.

Radiological examinations (skeletal and chest radiography, liver echography) and BS were performed with conventional techniques. The principal criteria for interpretation of BS results were as previously reported (Nicolini et al, 1989).

Hormone receptors were measured using the dextran-coated charcoal (DCC) method, and results were given in femtomoles per milligram (fmol $mg^{-1}$) of cytosol protein. The cut-off level was 3 fmol $mg^{-1}$.

### Statistical analysis

Median and range were evaluated from mastectomy to tumour marker increase and from tumour marker increase to relapse. Median survival was evaluated from the times of mastectomy and salvage treatment. All these parameters were analysed separately in patients treated 'early' who were or were not receiving therapy when they were suspected for relapse (subgroups a and b) and in patients treated 'not early' (group c).

Times from mastectomy to tumour marker increase, the lead time from the suspicion of relapse on the basis of tumour markers to the clinical and/or radiological signs of metastases between patients treated 'early' and not treated 'early' were compared using the unpaired *t*-test.

Overall survival curves from mastectomy and from salvage treatment of patients treated 'early' and not treated 'early' were generated using the Kaplan–Meier method (Kaplan and Meier, 1958). Differences between survival curves were tested with the Mantel–Haenzsel statistic. The Cox proportional hazards regression analysis (BMDP 2L, Department of biomathematics, University of California at Los Angeles, USA, revised 1990) was used to examine whether any variable was an independent predictor of survival. Variables selected for examination were age (continuous values) menopausal status, surgery, staging, treatment delivered 'early' and not delivered 'early'. The overall observation periods from salvage treatment and from mastectomy were 76 and 144 months, respectively, and the last death occurred within these intervals. Survival curves were drawn and interrupted 30 and 72 months from the beginning, i.e. after most events had occurred, and the statistical difference began to decrease progressively, probably because of the small residual samples (Figures 1 and 2).

As regards patients treated 'early', only OS curves of group a+b were compared with those of the group not treated 'early'. Following recent recommendations (Black and Welch, 1993) the mean lead time (3.4 months) observed in the 22 patients with conventional radiological examination-based diagnoses of metastases was subtracted from the time interval from salvage therapy to death for each one of the 28 patients treated 'early'; the resulting time intervals were used to build up the OS curve from salvage treatment for the 28 patients treated 'early' (Figure 2).

### RESULTS

### Clinical outcome

Thus far, 56 (15%) patients have died, but for four patients (three $N^-$ and one $N^+$) death was apparently due to reasons other than metastatic spread. Specifically, in three patients, the likely cause of death was another cancer (one pancreatic, one hepatic cancer in cirrhosis and one colorectal cancer), and in the fourth patient it was cirrhosis.

Disease recurrences occurred in 65 patients; 13 of these patients belonged to the group of 79 patients who withdrew from the follow-up protocol and two others were excluded as a result of rapidly progressive disease. Recurrences in the 50 patients followed were: at distant sites only, 34; at distant sites and locoregional, 16 (11 in the group treated 'early' and five in the group treated 'not early'). In 48 (96%) of these 50 relapsed patients with distant or distant and locoregional metastases, the increase in tumour marker preceded ($n$ = 47) or occurred contemporaneously ($n$ = 1) with the diagnosis made by standard investigations.

### Reliability of tumour markers and imaging techniques

The increase in one or more of the three markers preceded the appearance of the clinical and/or radiological signs of relapse in 47 of the 50 (94%) patients evaluated. However, in 5 (10.6%) of these

© Cancer Research Campaign 1997

47 patients, an equivocal BS finding occurred earlier, and the bone abnormalities were confirmed by computerized tomography or skeletal radiography directed at the tracer avid areas on BS 4.5 ± 4 (mean ± s.d.) months after CE or PI of one or more markers had occurred. In two of the three remaining patients, no increase in tumour marker was found at the time of relapse. In one of these cases, liver echography and, in the other, chest radiography were the first pathological finding. In the last case, the increase in one or more markers was contemporaneous with the appearance of radiological signs of metastases. In the 200 non-relapsed patients followed-up at 4-month intervals, the specificities of CEA, TPA, Ca15-3 and the CEA–TPA–Ca15-3 panel were 97%, 93%, 98% and 89% respectively. In the 119 remaining patients who underwent follow-up visits at 6-month intervals, the specificities were 97.5%, 96%, 99% and 93% respectively. With regard to the imaging techniques, the specificities of skeletal radiography, liver echography and BS were 97%, 98% and 59% respectively.

### Evaluation of 'early' salvage treatment

Table 3 shows in subgroups a, b and in group c the median and range of the time from mastectomy to tumour marker increase and from an increase in tumour marker to the definite radiological and clinical signs of metastases. In addition, it shows survivals from mastectomy and salvage therapy. The time from mastectomy to tumour marker increase was not significantly different between patients treated 'early' (subgroups a and b) and patients treated 'not early' (group c). In subgroups a and b, the mean lead time from tumour marker increase to relapse was significantly longer ($P < 0.001$; unpaired *t*-test) than that in group c. In the patient groups treated 'early' (subgroups a and b), median survivals from mastectomy and from salvage treatment were 60, 78.5 and 24.5, 31.5 months respectively. In group c, the median survivals from mastectomy and from salvage treatment were 44.5 and 15 months respectively.

Figure 1 shows the survivals from mastectomy in patients treated 'early' (group a+b) and patients not treated 'early' (group c). The two groups show similar survivals in the first 30 months. Thereafter, the decrease in survival appears to be more rapid in the patients not treated 'early'. In fact, 72 months after mastectomy, the fraction of survivors is 42.9% in patients treated 'early' and 22.7% in patients not treated 'early'. The difference is statistically significant ($P = 0.041$).

Figure 2 shows survival from salvage therapy in the same two groups. In both groups, the decrease in survival is progressive and linear but it is faster in group c. In fact, after 30 months of observation, the survivals are 42.9% in the 'early' treated group and 13.6% in the group treated later. The difference between these two values is statistically significant ($P = 0.045$). Using the Cox proportional hazards regression analysis, only early delivery of treatment proved to be a highly significant variable.

### DISCUSSION

Thus far, clinical studies that fail to find clear benefits from post-operative monitoring of breast cancer patients have been performed using conventional means (i.e. physical examination, routine laboratory and/or radiological examinations) and have excluded a role for tumour markers (Adair et al, 1974; Bishop et al, 1979; Horton, 1984; Ciatto et al, 1985; Andreoli et al, 1987). Two recent trials (Dixon et al, 1993*a* and *b*) have shown a role for tumour markers in guiding therapy and in the monitoring of the response to treatment of metastatic breast cancer patients. Furthermore, the observation that the growth rate of distant metastases is faster than in the primary tumour (Tubiana and Koscienly, 1988) suggests that an 'early' detection and treatment of distant metastases might be helpful. Our study used for the first time the measurement of a sensitive tumour marker panel for 'early' detection of distant metastases. This approach was useful as 47 (94%) of the 50 relapsed patients were detected 'early', i.e. before the appearance of radiological and/or clinical findings. This allowed the initiation of 'early' salvage treatment. In relapsed patients subjected to 'early' treatment, the lead time from the increase in tumour marker to the appearance of radiological signs of metastases was significantly prolonged ($P < 0.001$; unpaired *t*-test). During this interval, no or only minor symptoms occurred. As the increase in tumour marker is likely to warn of the risk of progression to overt metastatic disease, the prolongation of this time suggests that 'early' treatment in responsive patients slows or temporarily halts disease progression so as to delay the appearance of radiological signs. In patients treated 'early', median survival from salvage treatment and from mastectomy was prolonged when compared with that of patients treated only after metastases were ascertained by radiological means. The prolongation of the former interval might be considered misleading and due to the beginning of salvage treatment 'early' (Horton, 1984; Ciatto et al, 1985; Tomin and Donogan, 1987; Rutgers et al, 1989; Del Turco et al, 1994; Givio, 1994). This is the so-called lead time bias. Nevertheless, the prolongation of median survival from mastectomy, i.e. a stable end point, suggests that in patients treated 'early' and in responsive patients the prolongation of the interval from the time of tumour marker increase to the definite signs of metastases really does prolong both median survivals.

These concepts are confirmed by comparison of the survival curves from both the time of mastectomy and from salvage treatment. In fact the difference between the two curves from mastectomy is statistically significant, and that between the two curves from salvage therapy attains statistical significance even after the subtraction in the 28 patients treated 'early' of the mean lead time observed in the 22 patients treated not 'early'. As to the length bias that pertains to comparisons unadjusted for the rate of progression of disease (Black and Welch, 1993), it is not likely to affect the results of the study. In the groups a+b and c, the main prognostic indices were similar (Table 2). These findings are apparently in contrast with those so far reported in clinical studies with regard to the use of the post-operative follow-up of breast cancer patients. However, in most of these studies, tumour markers were not used at all or were not used with precise criteria to define the 'early' detection of distant metastases (Adair et al, 1974; Bishop et al, 1979; Horton 1984; Ciatto et al, 1985; Andreoli et al, 1987; Tomin and Donogan, 1987; Rutgers et al, 1989; Del Turco et al, 1994; Givio 1994). In our study, the criteria used for interpreting tumour marker levels together with the history and routine laboratory examinations at each follow-up visit kept the number of false-positive diagnoses of distant metastases to a minimum, simultaneously leading to a great saving in the number of radiological examinations.

Recently, some advantages of the 'early' detection of recurrences by measurement of tumour markers and the early use of hormonal therapy for treatment of low tumour burdens have been reported in the management of cancers other than breast. In fact, in a meta-analysis on the post-operative follow-up of colorectal cancer patients, studies with intensive follow-up that included serial serum

 © Cancer Research Campaign 1997

CEA assays revealed a higher rate of detection of asymptomatic tumours and radically resectable recurrences as well as a 9% better 5-year survival than did those series with minimal or no follow-up (Bruinvels et al, 1994). Similarly, a longer survival of prostate cancer patients is reported when hormonal therapy is initiated early in locally advanced disease (Einstein, 1995). In these patients, serial serum PSA determinations contribute to the 'early' detection and lower tumour burden of primary tumour.

Data from this study in breast cancer patients suggest that 'early' salvage therapy can delay disease progression of relapsed patients and that tumour markers play an important role for this purpose. In fact, determination of appropriate circulating tumour markers allows a simple and adequate follow-up as well as an 'earlier' identification of relapsed patients. We hope that this report will stimulate randomized prospective trials on the effect of early salvage treatment of breast cancer patients.

### ACKNOWLEDGEMENT

We thank Professor B Shapiro from Michigan University, Ann Arbor, MI, USA, for careful revision of the manuscript.

### REFERENCES

Adair F, Berg J, Joubert L and Robbins GF (1974) Long-term follow-up of breast cancer patients: the 30-year report. *Cancer* **33**: 1145–1150

American Joint Committee on Cancer Staging and End-Results Reporting (1983) *Manual for Staging of Cancer*, 2nd edn. Beahrs OH and Myers MA. (eds), pp. 127–133. Lippincott: Philadelphia

Andreoli C, Buranelli F, Campa T, Costa A, Magni A, Pizzichetta M and Ciatto S (1987) Chest X-ray survey in breast cancer follow-up. A contrary view. *Tumori* **73**: 463–465

Bishop HM, Blamey RW, Morris AH, Rose DH, Preston B, Lane J and Doyle PJ (1979) Bone scanning: its lack of value in the follow-up of patients with breast cancer. *Br J Surg* **66**: 752–754

Black WC and Welch HG (1993) Advances in diagnostic imaging and overestimations of disease prevalence and the benefits of therapy. *N Engl J Med* **328**: 1237–1243

Bruinvels DJ, Stiggelbout AM, Kievit J, Van Houwelingen HC, Habbema JD and Van De Velde CJ (1994) Follow-up of patients with colorectal cancer. A meta analysis. *Ann Surg* **219**: 174–182

Ciatto S, Rosselli Del Turco M, Pacini P, Mustacchi G, Simonis M, Sismondi P, Giardina G, Belsanti V, Aristei C, Molino AM, Capelli MC, Azzini V, Di Costanzo F, Buzzi F, Murgo R, Punzo C, Gosso P and Locatelli E (1985) Early detection of breast cancer recurrences through periodic follow-up. Is it useless? *Tumori* **71**: 325–329

Del Turco MR, Palli D, Cariddi A, Ciatto S, Pacini P and Distante V (1994) Intensive diagnostic follow-up after treatment of primary breast cancer. A randomized trial. *JAMA* **271**: 1593–1597

Dixon AR, Jackson L, Chan SY, Badley RA and Blamey RW (1993*a*). Continuous chemotherapy in responsive breast cancer: a role for tumour markers? *Br J Cancer* **68**: 181–185

Dixon AR, Price MR, Hand CV, Sibley PEC, Selby C and Blamey RW (1993*b*) Epithelial mucine core antigen (EMCA) in assessing therapeutic response in advanced breast cancer – a comparison with CA 15.3. *Br J Cancer* **68**: 947–949

Einstein AB Jr (1995) Hormonal therapy for prostate cancer. When to use it. *Cancer Control* **2**: 32–36

Horton J (1984) Follow-up of breast cancer patients. *Cancer* **52**: 790–797

International Union against Cancer, Committee on TNM Classification (1974) *TNM Classification of Malignant Tumours*, 2nd edn. International Union against Cancer: Geneva

Kaplan EL and Meier P (1958) Non parametric estimation from incomplete observation. *J Am Stat Assoc* **53**: 457–481

Nicolini A, Carpi A, Di Marco G, Giuliani L, Giordani R and Palla S (1989) A rational postoperative follow-up with carcinoembryonic antigen, tissue polypeptide antigen, and urinary hydroxyproline in breast cancer patients. *Cancer* **63**: 2037–2046

Nicolini A, Colombini C, Luciani L, Carpi A and Giuliani L (1991*a*) Evaluation of serum CA 15-3 determination with CEA and TPA in the post-operative follow-up of breast cancer patients. *Br J Cancer* **64**: 154–158

Nicolini A, Carpi A and Tibaldi C (1991*b*). The postoperative management of breast cancer patient: new concepts. In *Progress in Clinical Oncology*, Carpi A, Sagripanti A and Mittermayer CH. (eds), pp. 187–203. Sympomed Medical Publisher: Munchen

Rutgers EJTH, Van Slooten EA and Kluck HM (1989) Follow-up after treatment of primary breast cancer. *Br J Surg* **76**: 187–190

The Givio Investigators (1994) Impact of follow-up testing on survival and health-related quality of life in breast cancer patients. A multicentric randomized controlled trial. *JAMA* **271**: 1587–1592

Tomin R and Donogan WL (1987) Screening for recurrent breast cancer: its effectiveness and prognostic value. *J Clin Oncol* **5**: 62–67

Tubiana M and Koscienly S (1988) Kinetics, growth rate and the natural history of breast cancer. The Heuson Memorial Lecture. *Eur J Cancer Clin Oncol* **24**: 9–14

© Cancer Research Campaign 1997